# Melatonin as a master regulator of cell death and inflammation: molecular mechanisms and clinical implications for newborn care

**DOI:** 10.1038/s41419-019-1556-7

**Published:** 2019-04-08

**Authors:** Anna Tarocco, Natascia Caroccia, Giampaolo Morciano, Mariusz R. Wieckowski, Gina Ancora, Giampaolo Garani, Paolo Pinton

**Affiliations:** 1 Department of Morphology, Surgery and Experimental Medicine, Section of Pathology, Oncology and Experimental Biology, Laboratory for Technologies of Advanced Therapies (LTTA), University of Ferrara, Ferrara, Italy; 2 Neonatal Intensive Care Unit, University Hospital S. Anna Ferrara, Ferrara, Italy; 3 Maria Cecilia Hospital, GVM Care & Research, 48033 Cotignola, Ravenna Italy; 4 Department of Biochemistry, Nencki Institute of Experimental biology, 02-093 Warsaw, Poland; 5 Neonatal Intensive Care Unit, Infermi Hospital Rimini, Rimini, Italy

## Abstract

Melatonin, more commonly known as the sleep hormone, is mainly secreted by the pineal gland in dark conditions and regulates the circadian rhythm of the organism. Its intrinsic properties, including high cell permeability, the ability to easily cross both the blood–brain and placenta barriers, and its role as an endogenous reservoir of free radical scavengers (with indirect extra activities), confer it beneficial uses as an adjuvant in the biomedical field. Melatonin can exert its effects by acting through specific cellular receptors on the plasma membrane, similar to other hormones, or through receptor-independent mechanisms that involve complex molecular cross talk with other players. There is increasing evidence regarding the extraordinary beneficial effects of melatonin, also via exogenous administration. Here, we summarize molecular pathways in which melatonin is considered a master regulator, with attention to cell death and inflammation mechanisms from basic, translational and clinical points of view in the context of newborn care.

## Facts

- Melatonin is a ubiquitous molecule with natural and powerful antioxidant proprieties and administration of exogenous melatonin is safe
- Melatonin exerts anti-inflammatory effects mainly by inhibiting inflammasome activation
- Melatonin exerts its antiapoptotic activities mainly by blocking caspase 3 cleavage and mPTP opening
- “Oxygen radical diseases of neonatology” refers to the oxidative stress that has a leading role in the pathogenesis of neonatal morbidities and pathologic conditions

## Open questions

- How endogenous melatonin contrast the oxidative stress that has a leading role in the pathogenesis of neonatal morbidities and pathologic conditions?
- Which are the intracellular targets of melatonin?
- How could melatonin improve the treatment of neonatal disease?
- What factors ultimately determine the melatonin efficacy as an adjunctive treatment in sepsis, chronic lung disease and hypoxic–ischemic encephalopathy of the term and preterm infants

## Introduction

Melatonin (*N*-acetyl-5-methoxytryptamine) is a ubiquitous molecule present in nature that carries out many functions^1^, manifesting enormous versatility and diversity. More commonly known as the sleep hormone, melatonin also has antioxidant, anti-inflammatory, antiapoptotic, and many other crucial properties^2,3^. In mammals, this multitasking indolamine is synthesized in the pineal gland in a circadian manner in response to the photoperiodic information received via the retinohypothalamic pathway^4,5^. It is directly released into the bloodstream, where it is distributed to all tissues^6,7^. Melatonin has two important functional groups that determine its specificity and amphiphilicity: the 5-methoxy group and the N-acetyl side chain. In particular, due to its amphiphilic characteristics, melatonin can diffuse and easily cross all morpho-physiological barriers, such as the placenta or the blood–brain barrier^8,9^, and it can enter all cells of the body, influencing the function of a variety of tissues^10^. Pineal synthesis is timed by the suprachiasmatic nucleus of the hypothalamus, depending on the light–dark cycle over a 24-h period^5^. Melatonin is mainly produced during the dark phase, and the maximal plasma concentration of this serotonin-derived hormone usually occurs 4–5 h after darkness onset^11^. Light stimulus activates melanopsin breakdown in retinal photoreceptive ganglion cells that, via the retinohypothalamic pathway, induce the inhibition of melatonin synthesis^12^; as a consequence, during the daily light period, its level is low or even undetectable^13^. Throughout life, melatonin levels tend to be significantly reduced. In the blood, once secreted from the pineal gland, melatonin is usually bound to albumin, metabolized to 6-hydroxymelatonin by cytochrome P-450 isoforms and conjugated, in the liver, to produce the principal urinary metabolite, 6-sulfatoxy-melatonin, which is finally eliminated through the kidney^14^. However, melatonin is not exclusively produced in the pineal gland, but it is also locally synthesized in several cells and tissues, such as the retina, the gastrointestinal tract, and the innate immune system^10^. The synthesis in extrapineal sites presumably does not follow circadian rhythms, except for the retina, and mainly works as a local antioxidant^15,16^.

**Fig. 1:**
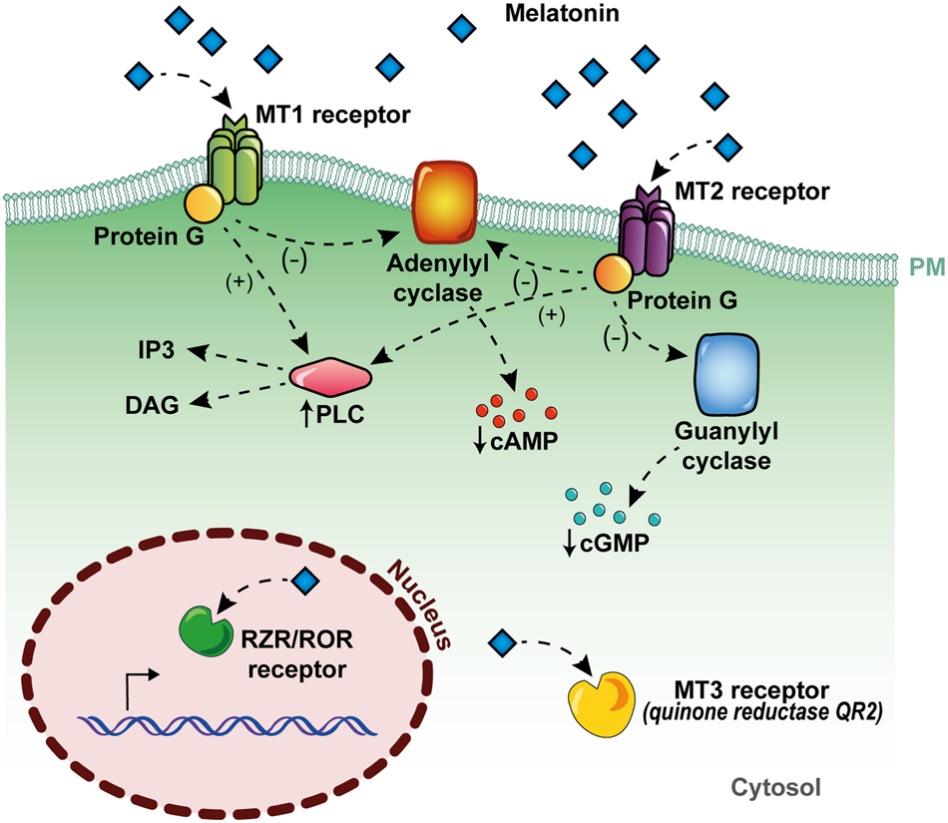
Mechanisms of action of melatonin. Melatonin can exert its effects by acting through receptor-independent mechanisms, which involve the direct interaction of melatonin and other molecules, and they are mainly related to its antioxidant and radical scavenging action (**a**). As any other hormone, melatonin can also act through specific cellular receptors, by membrane melatonin receptors, called MT1 and MT2, which are seven transmembrane-spanning proteins belonging to the G-protein-coupled receptor (GPCR) superfamily, by the cytosolic enzyme QR2 (also called MT3), or through the nuclear receptors RZR/ROR (**b**)

Studies have proposed that mitochondria are the primary sites of melatonin synthesis^17^. Mitochondria are major sources of free radicals, and in addition to being commonly used to treat disoriented circadian clocks due to jet lag and other disturbances (i.e., sleep inefficiency)^18^, melatonin has been widely used as an antioxidative therapy^19^ and its use dates back to 1993 (refs. ^20,21^). The direct antioxidant and free radical scavenging properties of melatonin are mainly due to its electron-rich aromatic indole ring, which makes it a potent electron donor that can significantly reduce oxidative stress^3,22^. Over this direct action, melatonin can further activate melatonin (MT) 1 and MT2 receptors (Fig. 1), upregulating antioxidative defensive systems by increasing the expression or activity of antioxidant enzymes such as superoxide dismutase and glutathione peroxidase^23^.

MT1 and MT2 are seven transmembrane-spanning proteins belonging to the G-protein-coupled receptor (GPCR) superfamily^24^, which exhibit high-affinity binding and could be activated at low concentrations of melatonin (pM–nM)^25^. Furthermore, a third melatonin-binding site (MT3) has been characterized and identified as the cytosolic enzyme quinone reductase 2 (QR2), a known detoxifying enzyme that reduces menadione and other quinones^26,27^. Moreover, melatonin is also a ligand for nuclear receptors, such as retinoid orphan receptors (or retinoid Z receptors), ROR/RZRα, and ROR/RZRβ^28–30^ (Fig. 1).

## Pathophysiological processes where melatonin plays important roles

### Antiapoptotic activity

**Fig. 2:**
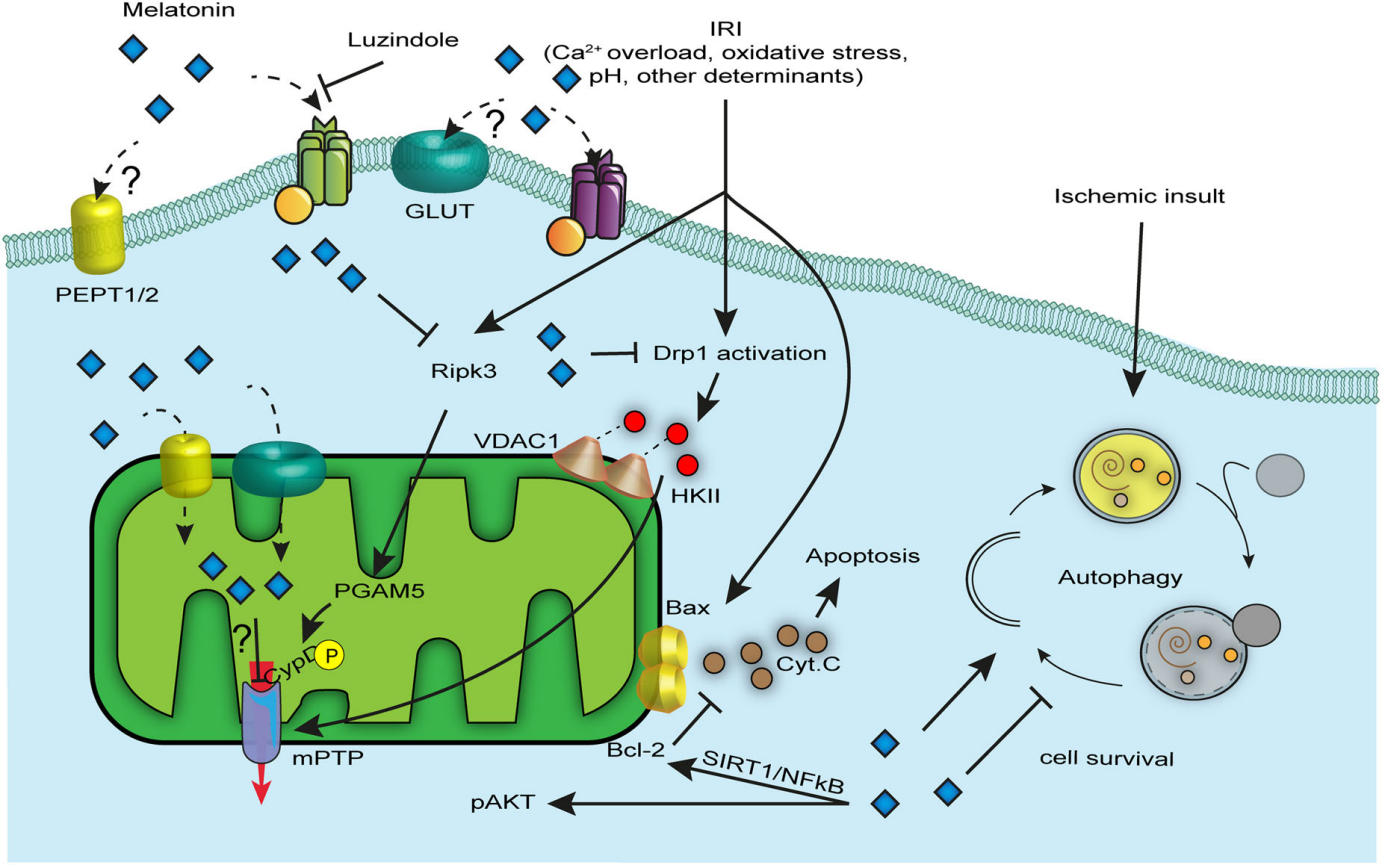
Antiapoptotic mechanisms operated by melatonin. Endogenous levels of melatonin and exogenous administration confer to injured cells protection from many cell death forms including apoptosis, necroptosis, mPTP-driven cell death, and autophagy. Melatonin is high cell permeable and its beneficial effects are mediated by both MT1/2-dependent and MT1/2-independent mechanisms. Once in the cytoplasm it blocks the Ripk3 cascade, Drp1 activation, and Bax-dependent cytochrome *c* (Cyt. *C*) release caused by external insults; as a result, the cell receives pro-survival signals. Melatonin localizes also in mitochondria where PEPT1/2 and GLUT channels are postulated to be new transporters of this hormone in the organelle. In mitochondria, melatonin modulates mitochondrial permeability transition pore (mPTP) opening and counteracts oxidative stress

Almost all the studies that monitored the melatonin-dependent antiapoptotic activities include disease models characterized by the presence of a hypoxic–ischemia (HI) event. HI describes a pathological condition in which an organ is subjected to harmful reduction in oxygen levels (hypoxia) and a deficit in blood supply (ischemia). This clinical picture entails the activation of several different pathways, and melatonin is known to modulate most of them, especially cell death. Mitochondria are organelles with key functions in the adaptive and maladaptive responses to brain injury^31^; indeed, they are strongly involved in cell death pathways, such as apoptosis, necrosis, and autophagy^32^, which cause an important portion of neuronal damage in the perinatal HI event (Fig. 2). In vitro experiments and neonatal animal models of HI suggested a very complex network of cell death cascades, highlighting a continuum from apoptosis to necrosis^33,34^. In other words, the coexistence of necrotic and apoptotic markers inside the same cell shape a heterogeneous phenotype characterized by a mixture of suicide program activation^33^. In the immature brain of rodents, the cell death continuum is more switched versus apoptosis; indeed, HI drives cell death through Bcl-2 family members^35,36^. Under these conditions, mitochondria permeabilize, and proapoptotic factors, such as cytochrome *c* (Cyt *C*) and the apoptosis-inducing factor (AIF), are released into the cytosol. Melatonin administration reversed this phenotype by adopting a compensatory mechanism aimed at increasing Bcl-2 protein expression, blocking Bax proapoptotic activity via the SIRT1/NF-kB axis with a consequent and significant inhibition of Cyt *C* release and the lack of apoptosome formation and caspase 3 activation^37^ (Fig. 2). The AIF pathway, together with increased levels of cleaved caspase 3, is recognized as the main inductor of apoptosis in the damaged brain of neonatal rodents^38^, even if their action becomes less pronounced once the brain matures^39^. Beneficial effects have also been reported by the use of melatonin in brain-injured mice 24 h after reperfusion with a selective action involving caspases 3 inhibition^40^. Nevertheless, melatonin also contributes to antiapoptotic activities via the Akt axis by preventing the decrease in pAkt and pBad levels upon HI injury^41,42^ (Fig. 2).

### Modulation of the permeability transition pore

Studies have reported that mitochondria permeabilize due to the mitochondrial permeability transition pore (mPTP) opening, a pathophysiological event that, under favorable conditions, causes an increase in permeability of the inner mitochondrial membrane and leads to mitochondrial depolarization, swelling, and the activation of the apoptotic and necrotic pathways^43^; for these reasons, mPTP is widely considered as the final step of cell demise^44^. Even though the exact structure of the mPTP, as well as its assembly kinetics, is still unknown, interesting findings suggested that dissociation of F_1_F_O_ ATP synthase dimers^45^ and the c subunit pore-forming part^46^ have an important role in mitochondrial permeability transition (mPT) and constitute a valid therapeutic approach in pathologies subjected to widespread cell death^47,48^. It is no coincidence that melatonin, in addition to having a plethora of beneficial effects, executes neuroprotection by modulating mPTP activity^49,50^. Indeed, melatonin is highly permeable to cell membranes and easily crosses the blood–brain barrier^51^, ensuring a good therapeutic profile for brain diseases, and is also able to accumulate into mitochondria, probably via GLUT/SLC2A and PEPT1/2 carriers^52,53^. Studies have shown how its use in mitochondria isolated from rodent brains and subjected to Ca^2+^-induced mPTP conferred protection from mitochondrial swelling and membrane depolarization^49^ and prevented Cyt *C* release and cardiolipin peroxidation^54^ via mPTP inhibition (Fig. 2). Moreover, the benefits of melatonin are appreciable in mitochondria isolated from aged rodent brains where chronic treatment allows for antiapoptotic effects and increased cellular respiration as the young mitochondria counterparts. The exact nature of this modulation (direct or indirect) is still evolving. In 2004, Andrabi et al. claimed a direct inhibition of mPTP by melatonin^55^, but he never identified the target pore protein; instead, a more recent study revealed that melatonin-mediated mPTP inhibition would be highly dependent on the MT1 receptor as mitochondrial protective effects did not occur in the presence of luzindole compound, an MT blocking agent^56^. Although melatonin-dependent mPTP modulation has been widely described with concordant results, related molecular mechanisms have only been proposed. Zhou et al. proposed two mechanisms by which (i) melatonin pretreatment represents an interfering mechanism for Ripk3/PGAM5/CypD axis execution, desensitizing cells to necroptosis triggered by RipK3 activation, PGAM upregulation, and CypD phosphorylation^50^ in endothelial cells; and (ii) melatonin avoids mPTP opening and mitophagy-mediated cell death by suppressing mitochondrial fission following ischemia reperfusion injury that in turn restored bound VDAC1-HK2 (ref. ^57^), limiting cell death in the cardiac microvasculature. However, whether melatonin plays the same role in HI brain injuries is unknown.

### Modulation of autophagy

Autophagy is engaged in intracellular material recycling to sustain cell bioenergetics^58^. The knowledge of melatonin as an autophagy modulator derives mainly from HI studies^59–62^. The interplay between melatonin and autophagy is discordant; indeed, if some studies showed that the hormone is able to significantly downregulate autophagy in different disease models^63–65^, other reports failed to confirm those findings, providing evidence for an enhancement of the autophagic process upon melatonin treatment^66–68^. Even when considering the same disease picture, for instance, the perinatal HI event, studies have shown how melatonin could prevent^60^ or enhance^59^ the autophagic response to the pathologic insult (Fig. 2).

Regardless of how and in which way melatonin modulates autophagy, it has been confirmed as a very important and functional protective agent^69^.

### Melatonin as a potent and widespread anti-inflammatory agent

Several studies have shown that melatonin can regulate the activation of the immune system, reducing chronic and acute inflammation^70–73^.

**Fig. 3:**
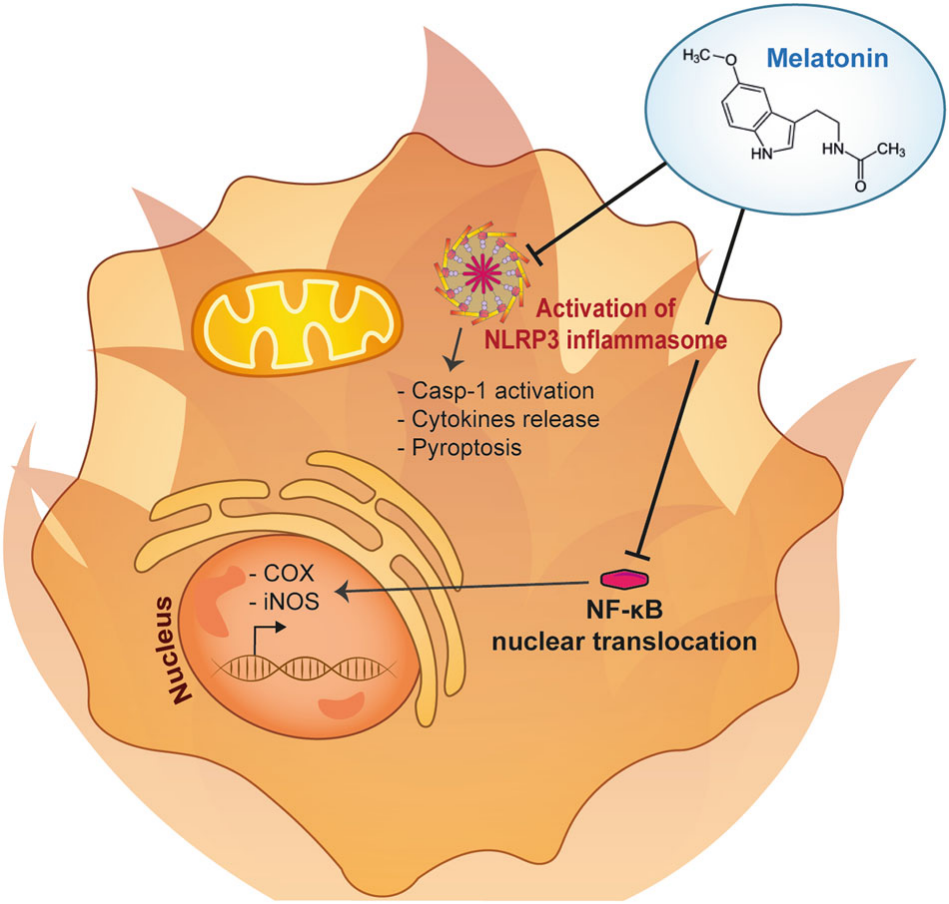
Anti-inflammatory effects of melatonin. Melatonin is mainly reported to possess anti-inflammatory properties by inhibiting inflammasome activation, thus inhibiting caspase-1 activation, cytokines release, and pyroptosis. In addition, melatonin can also inhibit the expression of the cyclooxygenase (COX) and inducible nitric oxide synthase (iNOS) by inhibiting nuclear NF-κB traslocation

Experimental and clinical data suggest that melatonin exerts its anti-inflammatory effects by modulating both pro- and anti-inflammatory cytokines in various pathophysiological situations^73,74^. Since different cytokines are associated with inflammatory diseases, wherein the balance between proinflammatory and anti-inflammatory molecules determines the clinical outcome to some degree, melatonin could modulate serum inflammatory parameters. In addition, melatonin inhibits the expression of cyclooxygenase (COX) and inducible nitric oxide synthase (iNOS)^75^ and limits the production of excessive amounts of prostanoids and leukotrienes and nitric oxide (NO), as well as other mediators of the inflammatory process, such as chemokines and adhesion molecules^73,76^ (Fig. 3).

In nonneuronal tissues, experimental studies have demonstrated that melatonin inhibits NO production induced by lipopolysaccharide (LPS) that induce the activation of the immune response^77^. The addition of melatonin, in a micromolar range, prevents LPS-induced iNOS expression in cultured rat endothelial cells and aortic rings^77^; this effect is not dependent on the activation of G-protein-coupled melatonin receptors but on nuclear factor-kappa B (NF-κB)^77^. Indeed, NF-κB triggers a cascade of molecular events, some of which may be potential key targets for the treatment of inflammation, and melatonin performs part of its anti-inflammatory functions by modulating nuclear NF-κB translocation^73,78,79^. Furthermore, melatonin is mainly reported to inhibit inflammasome activation^70,80^. Recently, Liu et al.^80^ demonstrated that this indole reduced LPS-induced inflammation and thus NLRP3 inflammasome formation in mouse adipose tissue by acting on the expression of inflammasome genes, including NLRP3, ASC, and thereby caspase-1 and IL-1β. In addition, the proinflammatory form of cells, called pyroptosis, was also strongly inhibited by melatonin^80,81^ (Fig. 3).

The anti-inflammatory properties of melatonin have also been extensively studied in models of cardiac sepsis^78,79,82^, where melatonin blunted the NF-κB/NLRP3 connection and activation^78,79,82^. To conclude, and accordingly to the data summarized here, melatonin is considered a potent molecule that has received increasing attention in the management of a large variety of diseases with an inflammatory etiology^7,70–73,79^ (Fig. 3).

### Analgesic and others modulatory roles of melatonin

The molecular pathways underlying the analgesic action of melatonin have not been completely addressed, and a plethora of mechanisms have been proposed, such as endorphin release from the pituitary gland^83^, modulation of the central GABAergic system^84^, and additional interaction with multiple receptor types. Those ligand-target bindings would include benzodiazepine, opioid, α(1)- and α(2)-adrenergic, serotonergic and cholinergic receptors^85^ and, notably, the fascinating issue of the anti-nociceptive role of melatonin seems to be mediated by MT1 and MT2 receptors themselves^86^^,^^87,88^. Studies have postulated that the cause may be ascribed to the reduction in the excitability of pain transmitting dorsal horn neurons via hyperpolarization due to MT1/2-melatonin binding^89^.

Among the effects provided by melatonin administration, the modulation of *N*-methyl-d-aspartate (NMDA) receptors and the conservation of myelin sheets in the central nervous system (CNS) are probably the least treated. NMDA receptors are sensitive to glutamate binding, the major excitatory neurotransmitter in the brain whose extracellular levels increase abnormally during insults, such as ischemia. Excessive release of glutamate and its binding with NMDA, α-amino-3-hydroxy-5-methyl-4-isoxazolepropionic acid (AMPA), or kainite (KA) receptors promotes excitotoxicity cell injury with a consequent activation of many molecular pathways, resulting in intracellular calcium (Ca^2+^) overload^90,91^, reactive oxygen species (ROS) production, mPT, and cell swelling with brain edema^92^. Patch-clamp experiments demonstrated how melatonin is able to modulate NMDA receptor activity by the drastic attenuation of their currents in neurons located in the spinal cord dorsal horn^93^. Focused experiments conducted in rat striatum synaptosome preparations have reported that melatonin inhibits the excitatory response in a partially Ca^2+^-dependent manner in which the direct effect on membrane hyperpolarization promoted intracellular Ca^2+^ influx reduction^94^. The involvement of melatonin is dose-dependent, and if at physiological concentrations, it inhibited NMDA-induced current. At higher dosages, it also participated in the modulation of AMPA-glutamate binding. It follows that melatonin exerts powerful protective mechanisms against oxidative damage and excitotoxicity mediated by glutamate receptors^95^.

Melatonin as a neuroprotective agent was also recommended in those pathologies involving white matter damage^96,97^. Indeed, researchers have reported a plethora of positive effects with different exposures of melatonin following CNS trauma, such as axonal regrowth and sprouting, conservation of a given thickness of myelin sheet in oligodendrocytes, and improved nerve maturation^96,97^.

As reported in the previous paragraphs, melatonin is a widespread potent molecule that can reduce cellular damage. Melatonin has recently received increased attention for its enormous potential in a wide range of different pathologies. In the next section, we discuss the use of melatonin for newborn care.

## Clinical utility in newborns

Oxidative stress has a leading role in the spectrum of neonatal disease processes, and it has been known for more than 160 years that hyperoxia has toxic effects on different organs. Saugstad^98^ in 2005 talked about the “oxygen radical disease of the newborn”: oxidative stress may involve different organs, often simultaneously, giving rise to different signs and different clinical manifestations.

**Fig. 4:**
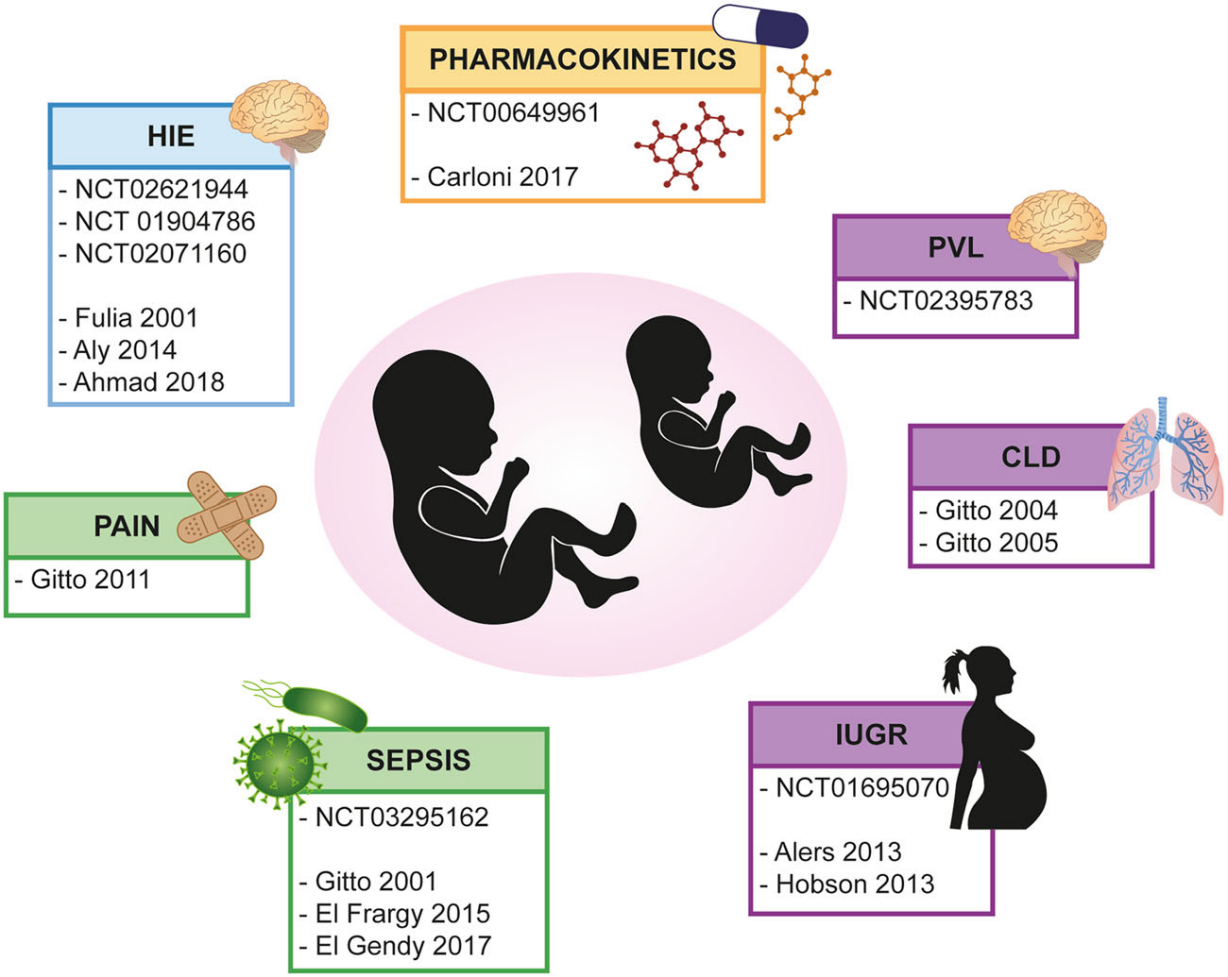
Clinical trial of melatonin in newborn care. Melatonin clinical trial in full term infants (blue), in preterm pathologies (purple) or both (green). In yellow are reported studies of pharmacokinetic. HIE hypoxic–ischemic encephalopathy, IUGR intrauterine growth retardation, CLD chronic lung disease, PVL periventricular leukomalcia

Babies at birth are naturally exposed to the hyperoxic challenge due to the transition from the hypoxic intrauterine environment (pO_2_ of 20–25 mmHg) to extrauterine life (pO_2_ of 100 mmHg). This transition naturally increases oxidative stress, but the gap is even more significant for infants that require resuscitation at birth with supplemental oxygen. Newborns are often exposed to oxygen therapies, have low antioxidant defenses despite high levels of toxic radicals, and are more susceptible to infections, especially if born prematurely^99^. Furthermore, inflammation is strictly correlated with oxidative stress in many conditions that affect newborns. To counteract free radical damage, therapeutic strategies in preclinical and clinical trials have tried to increase the antioxidant status of term and preterm infants, and melatonin, which is safe, nontoxic, and effective, has developed a leading role. Indeed, the efficacy of melatonin has been tested against the “oxygen radical diseases of newborn”, giving promising results^100–102^ (Fig. 4).

### Melatonin and sepsis

Sepsis is a major cause of morbidity and mortality in newborns, born both preterm and at term, with an incidence of 1–10 cases per 1000 live births and a mortality rate as high as 20% observed in very preterm newborns^103^. Although improvements in neonatal care have decreased the impact of early-onset sepsis in term infants, preterm babies remain at high risk for both early-onset and late-onset sepsis and their sequelae. According to the guidelines of the International Pediatric Sepsis Consensus Conference^104^, neonatal sepsis is defined as a clinical syndrome characterized by the presence of both infection and systemic inflammatory response syndrome (SIRS) and can cause severe neurological complications due to brain infection, as well as secondary hypoxemia resulting from septic shock, pulmonary hypertension and severe lung disease.

Clinically, SIRS includes respiratory symptoms, ranging in severity from mild tachypnea to respiratory failure, persistent pulmonary hypertension, irritability, lethargy, temperature instability, poor perfusion and hypotension, disseminated intravascular coagulation, poor feeding, vomiting and ileus. CNS involvement may presents with seizures, apnea, and depressed sensorium. It is accepted that bacterial infection induces sepsis via the production of endotoxins and the process is maintained by the inflammatory cascade and oxidative mechanisms that, once activated, operate independently from the presence of pathogens^103^. Intracellular redox changes are involved in the neonatal sepsis redox cycle and represent the main cause of cell dysfunction and mitochondria. As reported by Bajčetić et al. in 2014 (ref. ^105^), the immature innate immune system in neonates has a low capacity to generate ROS, so pro-oxidative processes in neonatal sepsis are limited to intracellular compartments of affected tissues. Moreover, neonatal cells appear to compensate for the infection-dependent mitochondrial dysfunction by extramitochondrial ATP production, and proliferating cells are particularly susceptible to apoptosis induced by oxidative stress^105^. This explains a higher incidence of long-term effects in neonatal sepsis survivors but also underlines the importance of different strategies for sepsis treatment both with antioxidant administration and pharmacologic inhibition of pro-oxidant pathways in addition to antibiotics.

Endogenous blood melatonin concentrations are higher in newborns with late-onset sepsis^106^, and its use as an adjuvant therapy in the treatment of sepsis is associated with improvement of clinical and laboratory outcomes^107^. In 2018, El-Gendy et al.^108^ published a study about the beneficial effect of melatonin in the treatment of neonatal sepsis that involved 40 septic neonates and concluded that the group who received melatonin had a significant improvement in clinical condition and serum parameters compared with the control group.

The review and meta-analysis of Henderson et al. enrolled 120 ill newborns from three different studies who were treated with melatonin as adjunctive therapies for sepsis. The results revealed statistically significant mean differences in C-reactive protein serum levels between groups at 24 h postadjunctive therapy with melatonin and a significant improvement of clinical condition in neonates from the intervention group compared to the control group within 3 days of therapy^109^. Based on clinicaltrial.gov, the recruitment phase of a randomized study aimed to assess the efficacy of melatonin as an adjuvant in the treatment of free radicals in septic preterm infants compared to the conventional approach alone was completed in Egypt (NCT03295162).

In conclusion, the use of melatonin as an adjunctive therapy for sepsis treatment significantly reduced inflammatory biomarkers and improved clinical conditions in neonates, but larger scale studies with higher validity are needed to demonstrate clear clinical benefits of the therapy^103,105,107–109^.

### Melatonin and preterm morbidity

As previously mentioned, oxidative stress has a leading role in the pathogenesis of preterm morbidities and pathologic conditions. Bronchopulmonary dysplasia (BPD), retinopathy of prematurity, intraventricular hemorrhage, and periventricular leukomalacia are only some examples^110^. Oxygen therapies used both in the delivery room and during hospitalization, immature organ development, and inflammatory/infective complications make the condition of preterm birth vulnerable to tissue injury^111,112^. Soon, melatonin could be used in preterm infants in the near future^113,114^, with the role of protective molecule against oxidative stress.

In addition, Gitto et al. in 2012 published a study suggesting a role for melatonin as an analgesic therapy during procedural pain in preterm babies, especially when inflammation is present. The Premature Infant Pain Profile score (PIPP score) was lower in 30 infants treated with common sedation plus melatonin during intubation and mechanical ventilation than in those treated with common therapy alone^115^. Likewise, the use of melatonin has rapidly spread; some authors proposed it for preterm babies affected by necrotizing enterocolitis^116^, for hemolytic hyperbilirubinemia-induced oxidative brain damage^117^, intrauterine fetal growth retardation^118–121^, chronic lung disease (CLD), and periventricular leukomalacia. Notably, melatonin did not reveal any side effects after single-dose administration to preterm babies born before 31 weeks of gestational age GA (for details, MIND phase II trial, NCT00649961).

Merchant et al.^122^ showed the difference between the pharmacokinetic profile in preterm babies and adults caused by immature liver and poor renal excretion and concluded that a 2-h infusion of 0.1 μg/kg/h increased plasma melatonin from undetectable to approximately peak adult concentration. The principal research objective of this multicenter double-blinded randomized placebo controlled trial was to determine the dose required to achieve physiological melatonin blood levels in preterm infants, similar to that of the mother and to define its pharmacokinetic profile in preterm infants. Additionally, in 2017, Carloni et al.^123^ showed the difference between pharmacokinetic profiles in premature newborns compared with adults, and despite the small sample size, they concluded that it is possible to obtain and maintain high serum concentrations using a single administration of melatonin repeated every 24/48 h.

### Periventricular leukomalacia

Periventricular leukomalacia (PVL) is a diffuse damage of the cerebral white matter that extends beyond the periventricular regions found predominantly in preterm infants.

PVL is due to three main mechanisms: hypoxia/ischemia of the vascular border zone, inflammation, excitotoxicity and free radical attack. Furthermore, diffuse lesion of PVL affects oligodendrocytes, which are the most vulnerable cells to injury with resulting myelin loss^124^.

Melatonin could be considered as the first candidate for clinical trials of neuroprotection in preterm infants, thanks to the peculiarity of easily crossing the placental barrier and its effect in improving myelin content and oligodendroglia cell maturation^125,126^. PRIMELIP, a multicenter therapeutic trial (NCT02395783), tested its neuroprotective action when administered in the immediate prepartum period in very preterm infants.

### Chronic lung disease

BPD, also known as CLD, is an important cause of respiratory illness in preterm newborns that results in significant morbidity and mortality. The epidemiology and pathology of BPD have changed over the past 50 years. “Old” BPD occurred in preterm infants with surfactant deficiency following respiratory distress syndrome (RDS). These infants required ventilatory support and high concentrations of supplemental oxygen therapies that induced lung damage with regions of atelectasis and regions of hyperinflation, epithelial injury, hyperplasia of airway smooth muscle, fibrosis, and pulmonary vascular hypertension. The improvement of neonatal RDS management as surfactant administration, antenatal glucocorticoid therapy, and less aggressive mechanical ventilation significantly decreased the morbidity and mortality of RDS and BPD in this population, shifting the demographics of BPD to earlier preterm infants (<29 weeks GA). As a consequence “new” BPD occurred at extremely low GA and is characterized by arrested alveolar-capillary development with larger, simplified alveoli; increased interstitial fibrosis and abnormal pulmonary vasculature; increased permeability with immature mechanisms for clearance of lung liquid; and recruitment of macrophages and neutrophils. These extremely low GA infants may not have surfactant deficiency or RDS but instead have early requirements for oxygen and ventilatory support due to multiple factors leading to “respiratory instability of prematurity”^127–129^. The management of RDS includes the prevention of hypoxemia and acidosis, the optimization of fluid management, the reduction in metabolic demands, the prevention of lung atelectasis and pulmonary edema, the reduction in lung damage due to aggressive mechanical ventilation, and the use of antioxidant strategies to minimize oxidant lung injury. Several investigators have reported that aggressive ventilatory strategies and oxygen therapy are the most important risk factors for lung disease. If oxygen radical-damaged tissues are present the premature lung is deficient in its antioxidant capacity. It follows that after oxygen injury, the inflammatory reaction develops, and IL1-β, IL-6, TNF-α and IL-8 are found in higher concentrations in babies who developed CLD^130,131^.

Furthermore, mechanical ventilation is a risk factor for cerebral inflammation and brain injury due both to the pulmonary inflammatory cascade, which migrates systemically to the brain, and to hemodynamic instability for the reduction in cardiac output and high pulmonary resistance caused by the distension of alveoli and compression of pulmonary capillaries^132^.

Gitto et al. published two studies in which approximately 100 newborns treated with melatonin as adjuvant antioxidant therapies of RDS were compared to 100 newborns conventionally treated. The authors concluded that melatonin treatment reduced proinflammatory cytokines in tracheobronchial aspirate, serum nitrite/nitare levels, and improved outcome because of its antioxidative actions^130^^,131^. Further studies are needed to investigate the possible use of melatonin as a preventive strategy of oxidative stress in preterm newborns. Thus, the preliminary results, i.e., the safety profile with high feasibility of administration, make melatonin a promising therapy for the prevention of BPD.

### Melatonin and asphyxiated term infants

Perinatal asphyxia refers to a condition during labor in which impaired gas exchange leads to fetal hypoxemia and hypercarbia. It is identified by fetal acidosis as pH < 7.0 and it is used in association with the neurological signs to evaluate term newborn at risk for brain injury in the perinatal period. The frequency of perinatal asphyxia is approximately 2–3/1000 live births^133^. The neurodevelopmental consequences of brain injury include death, cerebral palsy, severe intellectual disabilities, blindness, deafness, and a number of minor behavioral and cognitive deficits. HI can develop acutely or chronically during the prenatal (maternal factors such as hypotension, infection, hypoxia), perinatal (umbilical cord accidents, uterine rupture, placental factors), or postnatal period (shock, anemia, respiratory, or cardiac arrest)^134^.

In the pathological changes of neonatal HI encephalopathy (HIE), the time of injury and the time of treatment play an important role because of the cascading reaction process and cell changes. According to Wang, based on significant differences in the pathophysiology and biochemistry of brain tissues, it is possible to divide HIE into three phases, all of which have apoptosis or necrosis of nerve cells as the final outcome of brain damage^135,136^.

The primary energy failure phase (phase I) occurs 0–6 h after HI injury. Because of hypoxia and acidosis in tissues and organs, reduced myocardial contractility, decreased arterial blood pressure, and reduced cerebral blood flow, some cells undergo primary death based on the severity and duration of HI. Clinically, the treatment strategies during phase I utilize conventional methods: patients should be treated with hypothermia, free radical scavengers (e.g., melatonin, erythropoietin, coenzyme Q10), excitatory amino acid receptor blocking agents, and/or neuroprotective agents.

The secondary energy failure phase (phase II) occurs 6–72 h after HI, and the deterioration of oxidative metabolism has a leading role. Despite adequate oxygenation and circulation, excitatory neurotransmitters and free radicals continue to be released, phosphorus reserves are depleted, inflammatory factors are involved, and brain injury is substantial; as the time progresses, nerve cell apoptosis begins. This phase is marked by the onset of seizures, secondary to excitotoxic edema, cytokine accumulation, and more serious mitochondrial dysfunction. Therefore, the treatment strategies during phase II follow the treatments employed during phase I: babies should be treated with anti-inflammatory, neuroprotective, or nerve regenerating agents (e.g., nerve growth factor), and stem cell transplantation.

The injury repair or chronic inflammation phase (phase III) occurs days, months, and years after HI insult. Based on the severity of the disease, the duration of HI and the effects of prior therapeutic interventions, there are generally two outcomes: one involves recovery, where the damaged brain tissue enters the repair process and the surviving neurons and glial cells begin to differentiate, proliferate, and regenerate; in the other outcome, the injured tissue continues to deteriorate, and the mechanism of the persisting damage involves gliosis with the loss of support of neurotrophic factors, persistent inflammatory receptor activation, and changes in microglia and astrocytes that continue to release harmful cytokines, which promote neuronal death and axonal injury. Even in this case, the treatment should include anti-inflammatory agents, neuroprotective agents, or nerve regenerating agents (e.g., nerve growth factor) and stem cell transplantation. Once the patient’s condition has stabilized, a rehabilitation program should be planned for the patient as early as possible^135,137–139^.

The literature reports that melatonin serum levels increase after HI, such as the endogenous neuroprotective response to brain injury^135^. Thus, melatonin appears to be a good candidate for neuroprotection because of its safety profile and different protective effects, including ROS scavenging, excitotoxic cascade blockade, and modulation of neuroinflammatory pathways^140^. In addition, if melatonin is used in combination with hypothermia, the neuroprotective effects are greater than hypothermia alone^134,141–146^. In 2015, Aly et al.^147^ published a randomized trial in which the melatonin/hypothermia group, at 5 days of life, had fewer seizures and fewer white matter abnormalities on MRI. The limitation of that study was the small number of patients, but the authors concluded that compared with healthy neonates, the HIE group had increased melatonin, SOD, and NO concentrations, and the combination of melatonin to therapeutic hypothermia in infants with HIE can improve neurodevelopmental outcome at 6 months of age with effective effects in reducing oxidative stress in terms of NO and SOD serum level reduction. Another example comes from the studies by Fulia^148^ demonstrating that melatonin exerted protective actions by reducing malondialdehyde and nitrite/nitrate levels in newborn blood, improving survival. More recently, Ahmad et al.^149^ published a randomized control trial using hypothermia or hypothermia plus melatonin in 80 babies with HIE where melatonin improved survival rate.

Based on clinicaltrial.gov, a dose escalation study to evaluate the efficacy of enteral melatonin in infants with HIE is underway (NCT02621944). The primary outcome is to identify the maximum dose tolerated, and the second aim is to evaluate neurological outcome.

A phase 3 trial to test the benefits of melatonin treatment in association with hypothermia in infants with HIE was withdrawn prior to enrollment in 2015 (NCT01904786). Another phase 1 and 2 trial was completed in Egypt to examine the effect of combining melatonin with whole-body cooling on brain injury and the outcome of neonates following perinatal asphyxia (NCT02071160).

## Conclusions

Few conclusive results are available from clinical trials and from the literature (Fig. 4), despite encouraging data regarding melatonin as an adjunctive treatment in neonatal disease, particularly in term infants with HIE.

The goals of future clinical trials should be to establish the therapeutic range of melatonin dosage and the appropriate timing of administration to improve clinical condition and outcome.
